# Predicting CRISPR-Cas9 off-target effects in human primary cells using bidirectional LSTM with BERT embedding

**DOI:** 10.1093/bioadv/vbae184

**Published:** 2024-12-30

**Authors:** Orhan Sari, Ziying Liu, Youlian Pan, Xiaojian Shao

**Affiliations:** Department of Mining and Materials Engineering, McGill University, Montreal, QC, H3A 2B1, Canada; Digital Technologies Research Center, National Research Council Canada, Ottawa, ON, K1A 0R6, Canada; Digital Technologies Research Center, National Research Council Canada, Ottawa, ON, K1A 0R6, Canada; Digital Technologies Research Center, National Research Council Canada, Ottawa, ON, K1A 0R6, Canada; Department of Biochemistry, Microbiology and Immunology, Ottawa Institute of Systems Biology, University of Ottawa, Ottawa, ON, K1H 8M5, Canada

## Abstract

**Motivation:**

Clustered Regularly Interspaced Short Palindromic Repeats (CRISPR)-Cas9 system is a ground-breaking genome editing tool, which has revolutionized cell and gene therapies. One of the essential components involved in this system that ensures its success is the design of an optimal single-guide RNA (sgRNA) with high on-target cleavage efficiency and low off-target effects. This is challenging as many conditions need to be considered, and empirically testing every design is time-consuming and costly. *In silico* prediction using machine learning models provides high-performance alternatives.

**Results:**

We present CrisprBERT, a deep learning model incorporating a Bidirectional Encoder Representations from Transformers (BERT) architecture to provide a high-dimensional embedding for paired sgRNA and DNA sequences and Bidirectional Long Short-term Memory networks for learning, to predict the off-target effects of sgRNAs utilizing only the sgRNAs and their paired DNA sequences. We proposed doublet stack encoding to capture the local energy configuration of the Cas9 binding and applied the BERT model to learn the contextual embedding of the doublet pairs. Our results showed that the new model achieved better performance than state-of-the-art deep learning models regarding single split and leave-one-sgRNA-out cross-validations as well as independent testing.

**Availability and implementation:**

The CrisprBERT is available at GitHub: https://github.com/OSsari/CrisprBERT.

## 1 Introduction

The Clustered Regularly Interspaced Short Palindromic Repeats (CRISPR)-Cas9 system is a two-key-component system consisting of the target-specific CRISPR guide RNA (gRNA) and Cas9 endonucleases, where CRISPR single-guide RNA (sgRNA) identifies the target site to be cleaved by Cas9 endonuclease to achieve subsequence insertion or deletion of a fragment of DNA (Wiedenheft *et al.* 2012, Cong *et al.* 2013, Mali *et al.* 2013, Bak *et al.* 2018). In the CRISPR-Cas9 system, the gRNA (spacer) sequence needs to be complementary with its targeting DNA sequence, containing 20 nucleotides, which is followed by a three-nucleotide sequence called protospacer adjacent motif (Cong *et al.* 2013, Ran *et al.* 2013). The CRISPR-Cas9 system has been widely implemented in various species and cell types and has great potential for human therapeutics (Doudna and Charpentier 2014, Dever *et al.* 2016, Eyquem *et al.* 2017, Kurata *et al.* 2018).

Although the CRISPR-Cas9 system has become a powerful gene-editing tool, a major challenge for its effective application is to design/choose the optimal sgRNA, which has high on-target cleavage efficacy and low off-target effect (OTS). Indeed, not all sgRNAs would cut a target DNA with equal efficacy, i.e. different sgRNAs have different on-target efficiencies (Yan *et al.* 2018). Meanwhile, the Cas9 system scans the whole genome and possibly cuts unintended DNA sequences (off-targets) (Fu *et al.* 2013; Hsu *et al.* 2013). Thus, the off-target activity has been a major concern since the invention of the CRISPR-Cas9 system, especially for therapeutic and clinical applications.

To detect the off-target activities of sgRNAs genome-wide in a sensitive and unbiased way, several experimental techniques have been developed such as GUIDE-Seq (Tsai *et al.* 2015, Malinin *et al.* 2021), Digenome-Seq (Kim *et al.* 2015, 2021), SITE-Seq (Cameron *et al.* 2017), CIRCLE-Seq (Malinin *et al.* 2021), HTGTS (Frock *et al.* 2015, Hu *et al.* 2016), BLISS (Yan *et al.* 2017), and CHANGE-Seq (Lazzarotto *et al.* 2020). Among these techniques, the CRISPR-Cas9 system induces double-strand break (DSB) cleavage sites either in the purified genomic DNA or in living cells. In spite of their respective advantages and limitations, which have been widely reviewed (Tsai and Joung 2016, Tasan and Zhao 2017, Tsai 2018) and comprehensively benchmarked regarding the sensitivities and resource requirements, etc. (Yan *et al.* 2020), methods for detecting off-target activities of the Cas9 system are still labor-intensive, high-cost, and some are even difficult to operate. *In silico* models provide relatively rapid, low-cost alternatives to predict the off-target activities of sgRNAs, thus facilitating the optimized design of sgRNAs beforehand.

Up to now, various computational tools have been developed to facilitate the optimal sgRNA design. These works could be classified into three categories: (i) alignment-based scoring; (ii) hypothesis-driven testing, and (iii) machine learning model-based predictors. With increased data generated from the CRISPR community, machine learning, especially deep-learning-based models have become the main-stream. More recently, deep learning principles-based prediction systems have surpassed their competitors (Chuai *et al.* 2018, Lin and Wong 2018, Liu *et al.* 2019, Lin *et al.* 2020, Liu *et al.* 2020). Particularly, DeepCRISPR (Chuai *et al.* 2018) employed a deep convolutional denoising neural network-based autoencoder architecture to learn the deep representation of each sgRNA sequence and their associated epigenetic features, and further followed by a fully convolutional neural network (CNN) model for building the classifier. The autoencoder-based pre-training models on massive unlabeled sgRNA sequences in the whole genome help to capture sgRNA representations efficiently. AttnToMismatch_CNN (Liu *et al.* 2019) applied a transformer architecture with multi-heads attention modules to perform the encoding and decoding of each sgRNA and DNA sequence pair. CRISPR_Net (Lin *et al.* 2020) proposed a new sequence encoding scheme, which considered both mismatch and indels (i.e. insertions and deletions), and then connected by a recurrent convolutional network combining Inception-based CNN and bidirectional long short-term memory (BiLSTM) for learning the network classifier. These are three representations of off-target prediction studies using various advanced deep learning models. Several other studies were published during the preparation of our study, and they were more or less based on different combinations of CNN and recurrent neural networks (RNN) with different sequencing embedding approaches (Liu *et al.* 2020, Charlier *et al.* 2021, Zhang and Jiang 2022). In addition, for an overview of machine learning model or deep learning-based CRISPR sgRNA design tools, readers are referred to recent benchmarking studies (Wang *et al.* 2020; Sherkatghanad *et al.* 2023; Zhang *et al.* 2023).

Most of the current available deep learning models for CRISPR-Cas9 off-target predictions were trained on small sets of sgRNAs in various cell-lines and not evaluated on a large set of sgRNAs in human primary cells. In this study, we aim to develop a new deep learning model for CRISPR-Cas9 off-target predictions by exploring the performance of large-scale human primary cells. Specifically, we proposed a new stack encoding to encode the sgRNA–DNA pairs and adopted the Bidirectional Encoder Representations from Transformers (BERT) architecture for contextualized embedding followed by a conventional BiLSTM architecture for the deep learning model training. Our experiments demonstrated that the proposed new model outperformed existing deep learning models (including DeepCRISPR, CRISPR-Net, and AttnToMismatch_CNN) through single split and leave-one-sgRNA-out cross-validations as well as independent testing.

## 2 Methods

### 2.1 Benchmark dataset

#### 2.1.1 Cell-line datasets from DeepCRISPR

For comparison purposes, we collected the cell-line CRISPR-Cas9 off-target datasets from previous studies: DeepCRISPR (Chuai *et al.* 2018) and AttnToMismatch_CNN (Liu *et al.* 2019). The positive pairs were generated in multiple studies with different genome-wide off-target screening protocols across two cell lines: the HEK 293-related cell lines (18 sgRNAs) and K562T (12 sgRNAs). The positive pairs were the same between the DeepCRISPR and AttnToMismatch_CNN studies. However, the negative pairs were slightly differently generated by either Bowtie or Cas-OFFinder (Bae *et al.* 2014). Here, the negative set generated by Cas-OFFinder with up to six mismatched bases in each pair was selected. In total, 656 positive off-target sites and 169 557 negative off-target sites were collected.

#### 2.1.2 Primary cell dataset from CHANGE-Seq

We collected the human primary T-cell data generated by CHANGE-Seq (Lazzarotto *et al.* 2020), a recently developed *in vitro* genome-wide off-target cleavage site technique. In this dataset, 110 sgRNA targets across 13 therapeutically relevant loci were screened and 202 043 sgRNA–DNA pairs were measured. Among these pairs, 191 528 pairs contain only mismatches, i.e. no indels. In addition, when comparing the detailed pairs, 66 109 sequence-based redundant pairs (i.e. completely duplicate pairs regardless of the genomic positions) were removed. Thus, in total, 125 419 unique sgRNA–DNA pairs containing 27 410 positive and 98 009 negative off-target pairs were used.

#### 2.1.3 Amplification-free long-read sequencing data from nano-pore or Pacific Biosciences (long-reads OTS)

In this dataset, a set of 55 high-confidence sgRNA cleavage sites from three different sgRNA targeting HEK293 genomic DNA were obtained by two amplification-free long-read sequencing techniques including Pacific Biosciences’ single molecular read-time sequencing (SMRT-OTS) and Oxford Nanopore Technologies’ nanopore sequencing (Nano-OTS) (Höijer *et al.* 2020). To collect negative pairs corresponding to these positive pairs, we used Cas-OFFinder to find potential sgRNA–DNA mismatch pairs with mismatched bases ≤6. Finally, 480 negative pairs were identified.

### 2.2 Feature encoding and model components

#### 2.2.1 Stack encoding

Inspired by energy-based model of Alkan *et al.* (2018) and to mimic the energy configuration for the sgRNA and DNA sequence pairs, as this is important to form the sgRNA and DNA double strand, we proposed to use stack encoding to represent the sgRNA–DNA pair. Specifically, a two-base length-sliding window was adopted to extract the dimer pairs (or doublets) from a length of 23-base pair (bp) sgRNA–DNA double strand with a step of one base from 5' end to 3' end of the sgRNA. Since the sgRNA sequence or more broadly the DNA sequence consists of four nucleotides (A, C, G, T), there are 256 (4^4^) different types of dimers (“vocabulary sets”) that can be formed. Thus, the 23-bp length of the sgRNA–DNA sequence pair was converted to a vector of 22 dimers.

#### 2.2.2 Word embedding

The next step is to encode the discrete vector of 22 dimers into dense numeric features. We applied word embedding to map each dimer into a *d*-dimensional vector of floating point values. The word embedding, also known as distributed word representation, is an unsupervised learning algorithm that can capture both the semantic and syntactic information of words from a large unlabeled corpus. It would transform the dimer to low-dimensional numeric features and similar dimers would have a similar embedding feature. The embedding dimension is a hyper-parameter, which can be trained together with the deep learning architecture. We set the embedding dimension to 64 in the final model. This function was implemented using Keras embedding function in “TensorFlow” (Abadi *et al.* 2015).

#### 2.2.3 BERT embedding

The BERT model is a special type of transformer model (Devlin *et al.* 2019), where many encoders are stacked on top of each other. Encoder architectures are used for understanding the semantic meaning of tokens in a given vocabulary within natural language processing (NLP) tasks. These stacked encoder structures are proven to be effective in solving NLP tasks, but they are usually hard to interpret. We adopted the BERT model to perform a deep feature representation for the sgRNA–DNA pairs, as a contextualized embedding layer. For this purpose, we used a relatively small architecture, limiting the layer size and attention heads to six and eight, respectively. This architecture is smaller than the conventional BERT architectures, where the BERT BASE model has 12 layers and 12 heads (Devlin *et al.* 2019). The BERT model takes the same input as the word embedding model, i.e., a discrete vector of 22 dimers/doublets of the paired sgRNA and the DNA sequences. The embedding dimension was set to 64, thus the output of the BERT layer is a 64-dimensional continuous embedding vector. In this study, the BERT model was trained from scratch, and therefore pre-trained model was not used. The BERT architecture was implemented and maintained in the “Transformer” Python package, which is managed by the “HuggingFace” company.

#### 2.2.4 BiLSTM

An LSTM architecture is composed of many memory blocks. These memory blocks are able to integrate information from previous blocks and retain the important ones, while taking direct inputs from the data. To achieve that, each memory block has an input, output, and a forget gate. These gates determine which part of the input information should be stored, output, and for how long it should be stored, respectfully. The LSTM layers triumph over traditional RNN layers on issues such as better information management and avoiding exploding and/or vanishing gradients. Particularly, we used a BiLSTM network to extract the forward and backward information of the sgRNA–DNA sequence pairs. In the BiLSTM layer, a forward LSTM computes a representation ht→ of the sequence from left to right at every word t, and a backward LSTM computes a representation ht← of the same sequence in reverse. These two distinct networks use different parameters, and then the representation of a word ht = (ht→; ht←) is obtained by concatenating its left and right context representations (Luo *et al.* 2018). At the output of the BiLSTM layer, the forward and backward outputs of both LSTMs are combined together and concatenated.

### 2.3 Deep learning model

**Figure 1.**
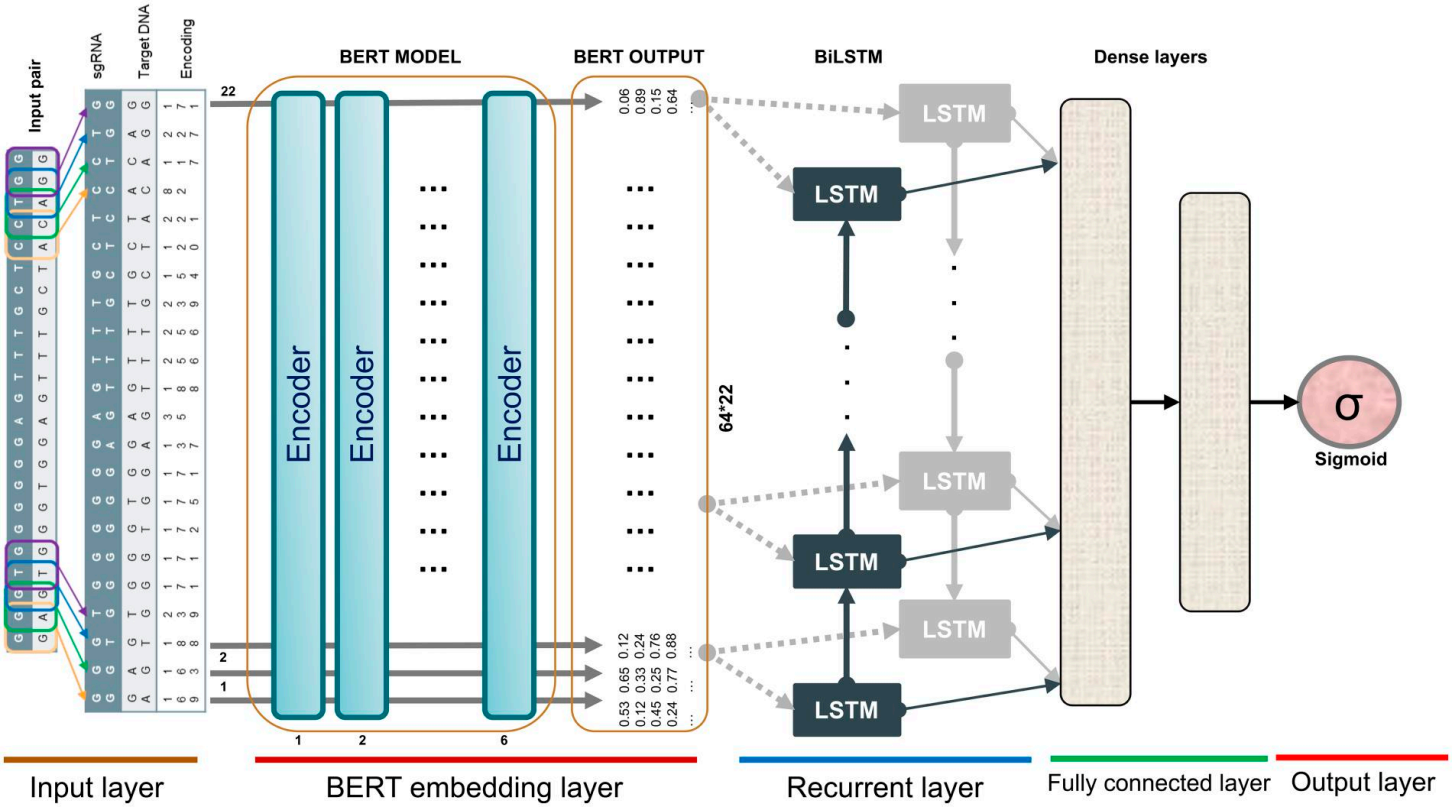
The CrisprBERT architecture. Stack encoding was used to encode the CRISPR-Cas9 sgRNA–DNA sequence pairs and followed by a BERT embedding layer. The complementary sequences to sgRNA sequences were used to match the target DNA sequences. The matched sequences were then used to form doublet stacks, which served as input to the model. After the BERT output for the deep feature representations, a traditional BiLSTM layer was used to build the recurrent neural network. Finally, the dense layers were connected to the output layer where sigmoid function was adopted to calculate the final probability of the classes.

The proposed new model in this study is illustrated in Fig. 1. For comparison purposes, the model that used BERT as the embedding layer was named “CrisprBERT,” and the one that used conventional word embedding was simply called “BiLSTM.” First, the encoding (Input layer) output is fed into the BERT embedding layer. The output of the BERT layer is 64-dimensional representation vectors, giving a 64×22 matrix. This is fed into the BiLSTM module (Recurrent layer). The concatenated output of the BiLSTM layer then goes into a series of dense layers. The final dense layer has a sigmoid activation for binary classification purposes. The architecture is implemented using “TensorFlow” (Abadi *et al.* 2015).

Overall, the proposed new model consists of the stack encoding input layer, BERT embedding layer, BiLSTM recurrent layer, and two dense layers as well as a sigmoid output layer.

### 2.4 Performance evaluations

We adopted k-fold cross-validation, a single-split cross-validation and leave-one-sgRNA-out methods to evaluate the models. The conventional k-fold cross-validation was used for parameter tuning of the models. The single-split cross-validation was then used to compare this model with others, by leaving 10% of the data as a validation set. Finally, for the leave-one-sgRNA-out strategy, one sgRNA and their associated sgRNA–DNA pairs were put aside for validation, and the remaining sgRNAs were used for training. In addition, a completely independent testing was performed to evaluate the generalization ability of the models. The training datasets and testing datasets were from different experiments—mainly from different cell types or different protocols.

The metrics for evaluating the performance of the model are Receiver Operating Characteristic-Area Under Curve (ROC-AUC) and Precision-Recall-Area Under Curve (PR-AUC), which are both widely used in classification problems. The ROC curve is plotted as the true-positive rate [TP/(TP + FN)] against the false-positive rate [FP/(FP + TN)] under a series of thresholds where TP is true positive, FN is false negative, FP is false positive, and TN is true negative. The precision–recall curve is plotted as precision [TP/(TP + FP)] versus recall [TP/(TP + FN)] under a series of thresholds. PR-AUC score is particularly suitable for assessing the performance of models on an imbalanced dataset. The higher the value of PR-AUC, the better the performance of the model in class imbalance problems. The value of ROC-AUC and PR-AUC is in (0, 1), where 1 indicates a perfect performance.

### 2.5 Experimental settings

The proposed CrisprBERT and BiLSTM models were implemented using Python 3.7 with TensorFlow (2.5.0) as the backend. All experiments were carried out on a computer with Intel (R) Core (TM) i9-12900H CPU @ 3.50 GHz, Ubuntu 24.04.1 LTS and 32 GB RAM, as well as one NVIDIA GeForce GTX 3080 Ti Laptop GPU with 16 GB of memory.

## 3 Results

### 3.1 Stack encoding doublets distribution

**Figure 2.**
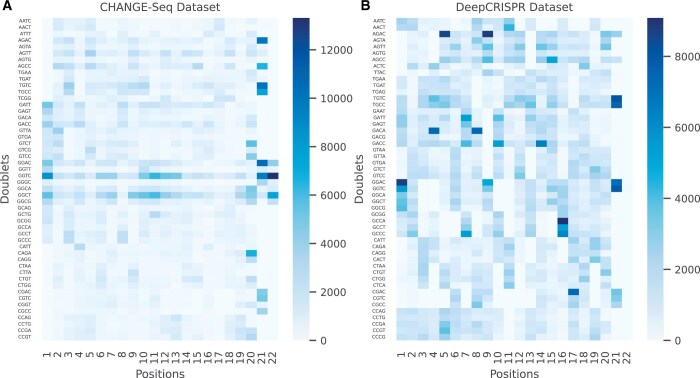
Heatmap of the top 50 doublets distribution in the CHANGE-Seq and DeepCRISPR datasets. (A) CHANGE-Seq dataset. (B) DeepCRISPR dataset. The frequency of the sgRNA–DNA doublets at each position (1–22 showed in the column label) was indicated with different colors—the darker, the larger of the frequency. Forty-one of these top 50 doublets were shared in both datasets. Mismatches of GG to other nucleotides were observed in the middle of the sgRNA sequences, particularly for the CHANGE-Seq dataset.

The top 50 doublets from both CHANGE-Seq and DeepCRISPR datasets were extracted, and their frequency distribution across different positions of the sgRNA–DNA pairs was calculated. Doublet distribution in both datasets showed the specificity of certain doublets in given positions (Fig. 2). Interestingly, 41 of the top 50 doublets were shared in both datasets, although different enrichments at different positions were observed. The top five observed doublets in CHANGE-Seq dataset are GG to TC, GG to CT, GG to AC, TG to TC, and AG to CC, whereas the top five doublets in DeepCRISPR dataset are AG to AC, TG to CC, TG to TC, GA to CC, and GG to AC. High frequency of TG to TC, TG to CC, GG to AC, and GG to TC doublets were consistently observed in the 21st position (i.e. last second positions) of both datasets. Besides the above enrichments, high frequencies of GG to TC and CT were also observed in the first and the middle of the sgRNA sequences in the CHANGE-Seq dataset, while GG to other nucleotides mismatches (e.g. GG to AC, TC, CT, and CG) were mainly observed at the first position in the DeepCRISPR dataset. Meanwhile, more diverse doublet mismatches were observed in the DeepCRISPR dataset than in the CHANGE-Seq dataset at other positions such as the AG to AC mismatches at positions 5 and 9, the GC to CA, CT, and CC at position 16.

### 3.2 Model comparisons

#### 3.2.1 Cross-validation

We first explored the influences of different encoding and embedding dimensions on the performance of the proposed CrisprBERT model. We compared singlet, doublet, and triplet encoding, as well as different embedding dimensions in the BERT layer. A cross-validation was performed on both DeepCRISPR and CHANGE-Seq datasets to evaluate the performance. The singlet and doublet encoding demonstrated comparable performance, while the triplet encoding exhibited reduced performance (Supplementary Fig. S1A and B). Similarly, results across various embedding dimensions indicated that the model with an embedding dimension of 64 performed slightly better than those with other parameters on both datasets (Supplementary Fig. S1C and D). Therefore, we opted for doublet encoding and an embedding dimension of 64 as the default setting for the CrisprBERT model.

After obtaining the optimal architecture and hyperparameters, the CrisprBERT and the simple BiLSTM models were compared with three different deep learning strategies previously published: DeepCRISPR, Attention_to_mismatch network, and CRISPR-Net.

**Figure 3.**
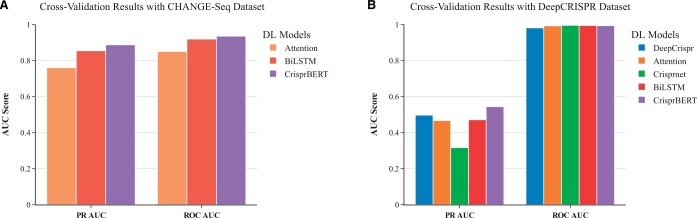
Cross-validation performance comparisons. Both PR-AUC and ROC-AUC were reported for the comparisons. (A) Performance on CHANGE-Seq dataset. (B) Performance on DeepCRISPR dataset.

The cross-validation performances for the CHANGE-Seq dataset were measured on three of the models: CrisprBERT, BiLSTM, and Attention_to_mismatch. However, we were unable to train the DeepCRISPR and CRISPR-Net models on this dataset as the source codes are not available. The validation for all three models was achieved using the same 10% of the dataset. Both BiLSTM and CrisprBERT outperformed the Attention_to_mismatch model (Fig. 3A). Specifically, the cross-validation ROC-AUC scores for Attention_to_mismatch, BiLSTM, and CrisprBERT were 0.85, 0.919, and 0.935, and the PR-AUC score for the models are 0.760, 0.854, and 0.887, respectively. These results imply that the simple sequence-based BiLSTM model with a proper doublet encoding can achieve similar results for off-target prediction compared to a denser and advanced neural network such as the Attention model. Meanwhile, the CrisprBERT model outperformed the BiLSTM model in both ROC-AUC and PR-AUC tests, indicating the BERT embedding has an advantage over the conventional word embedding.

The same cross-validation test was repeated using the DeepCRISPR dataset for both models. The PR- and ROC-AUC values for DeepCRISPR, Attention_to_mismatch, and CRISPR-Net models were taken from their respective studies. The comparison results are shown in Fig. 3B. Although all the models researched quite high ROC-AUC scores (i.e. ∼0.99), the PR-AUC scores are relatively small (i.e. around 0.5). Since this dataset is heavily imbalanced with much larger negative pairs than positive pairs, the PR-AUC is believed to be a more suitable metric. For this metric, the CrisprBERT again outperformed all other models, with a PR-AUC score of 0.544 (∼10% marginal increase over the other models). Meanwhile, the BiLSTM model remains comparable to DeepCRISPR and Attention_to_mismatch models.

Additionally, we explored the influences of sizes and data imbalance ratios in training data on the model performance. We therefore conducted cross-validation testing on subsets of the CHANGE-Seq dataset with different data sizes as well as subsets of the DeepCRISPR dataset with different imbalance ratios. As shown in the Supplementary Figs S2 and S3, the results demonstrated increased performances when the dataset size increased while a decreased performance when the data imbalance ratio between positive off-target and negative ones increased.

#### 3.2.2 Leave-one-sgRNA-out validation

**Figure 4.**
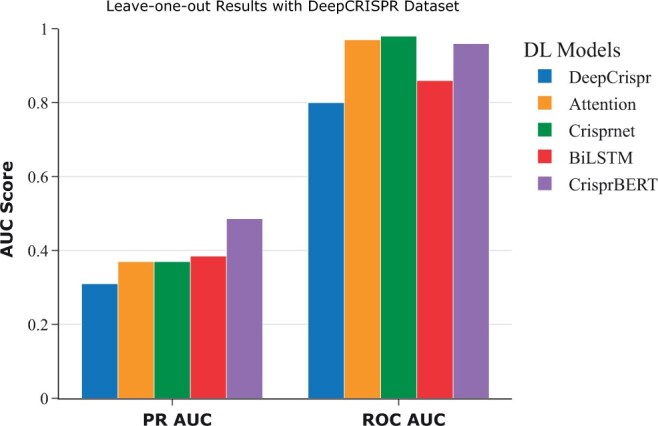
Leave-sgRNA-out testing performance comparisons. This testing was conducted on the well benchmarked DeepCRISPR dataset. The PR-AUC and ROC-AUC were measured on the all sgRNA–DNA pairs after completing the procedure of all sgRNAs.

To evaluate the generalization ability of the CrisprBERT model on predicting the off-targets of new (unseen) sgRNA, a leave-one-sgRNA-out experiment was performed to mimic the prediction performance of the model on new sgRNAs. In this particular test, a single sgRNA along with its corresponding off-target pairs were used for cross-validation and were left out of training. However, some sgRNAs have very few positive off-targets in both datasets (as low as one), which leads to statistical discrepancies, such as PR-AUC scores of 1 (Supplementary Fig. 4). Hence, some sgRNAs with very few positive off-target sequences were combined together to yield at least 30 positive off-target sequences. Specifically, we first sorted the sgRNAs based on the number of positive pairs. We then combined subset of the sgRNAs in a heuristic way (following the increased order of the number of positive pairs) to form the combined sgRNA sets, ensuring that each combined set includes at least 30 positive pairs. This leave-one-sgRNA-out validation was then repeated for all these combined sgRNAs, and the performance was measured over all the sgRNA–DNA pairs. We compared the performance of these two models with the other three models on the DeepCRISPR dataset only, where the ROC-AUC and PR-AUC of the DeepCRISPR, Attention_to_mismatch and CRISPR-Net models were extracted from the CRISPR-Net study. Figure 4 shows that CrisprBERT performed the best over other models regarding the PR-AUC metric, with a PR-AUC score of 0.486, which is more than a 10% marginal increase compared with other models. The BiLSTM model also showed a slight improvement in PR-AUC when compared with the other three models. In addition, it also achieved comparable ROC-AUC scores with other models.

#### 3.2.3 Independent testing

**Figure 5.**
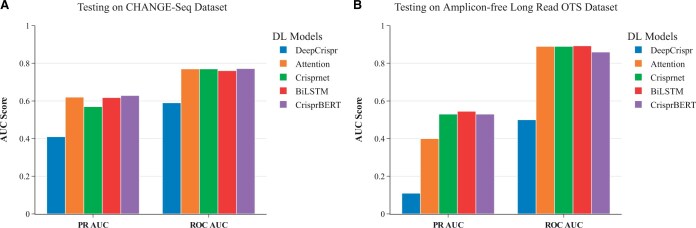
Independent testing performance comparisons. (A) Performance on CHANGE-Seq dataset; (B) Performance on long-read OTS dataset. BiLSTM and CrisprBERT models were trained on the DeepCRISPR dataset only and other models were from their respective study. Both testing datasets were not used for training in all the models.

To qualify the generalization capability of the models, independent tests were further conducted. It is particularly important to show the prediction performance on completely unseen data that are obtained from different experimental protocols, different sgRNAs, and different cell types. We trained BiLSTM and CrisprBERT on the DeepCRISPR dataset. For other comparison models, we used the released models from each study. We first tested them on the CHANGE-Seq dataset. As before, all indel sequences were removed from the CHANGE-Seq dataset. As the DeepCRISPR model required associated epigenomic features, we downloaded four epigenomic tables of the HepG2 cell line from ENCODE (Luo *et al.* 2020) and annotated the pairs in the CHANGE-Seq data. The results are shown in Fig. 5A. All models have comparable ROC-AUC or PR-AUC scores except the DeepCRISPR model, which showed lower scores. Specifically, CrisprBERT and Attention_to_mismatch performed similarly with CrisprBERT having a slightly higher PR-AUC score (i.e. 0.629, compared to 0.620 of Attention_to_mismatch). Both performed better than the BiLSTM and CRISPR-Net models. The CRISPR-Net model scored slightly less than the BiLSTM model. An almost identical pattern was observed with ROC-AUC scores.

Furthermore, all models were tested on another independent dataset, the long-read OTS dataset. For this dataset, when using the DeepCRISPR model, the corresponding epigenomic tables of the HEK293 cell line were extracted from ENCODE. A similar trend was observed in this test compared to the test on the CHANGE-Seq dataset (Fig. 5B). The CrisprBERT, BiLSTM, and CRISPR-Net models performed very similarly with respect to their PR-AUC and ROC-AUC scores, around 0.54 and 0.89, respectively. Attention_to_mismatch and the DeepCRISPR models performed a bit worse, achieving PR-AUC scores of <0.4.

### 3.3 Pooled datasets versus individual datasets

Finally, we explored whether training the model on integrated datasets would improve the performance of the CrisprBERT model through simply increasing the amount of training data. We performed the leave-one-sgRNA-out test on both DeepCRISPR and CHANGE-Seq datasets as well as the pooled dataset from these two for the BiLSTM and CrisprBERT models. Regarding the pooled dataset, the models followed the same protocol to produce the validation set. However, the training set size was increased by combining both datasets. We reported the global ROC-AUC and PR-AUC scores by merging all the individual datasets as well as the average ROC-AUC and PR-AUC scores of the individual leave-one-out sgRNAs. To reduce statistical variability, we made sure that every leave-one-out validation group had at least 30 positive off-targets. This implied merging some sgRNAs, which had very few positive off-targets.

#### 3.3.1 CHANGE-Seq dataset

**Figure 6.**
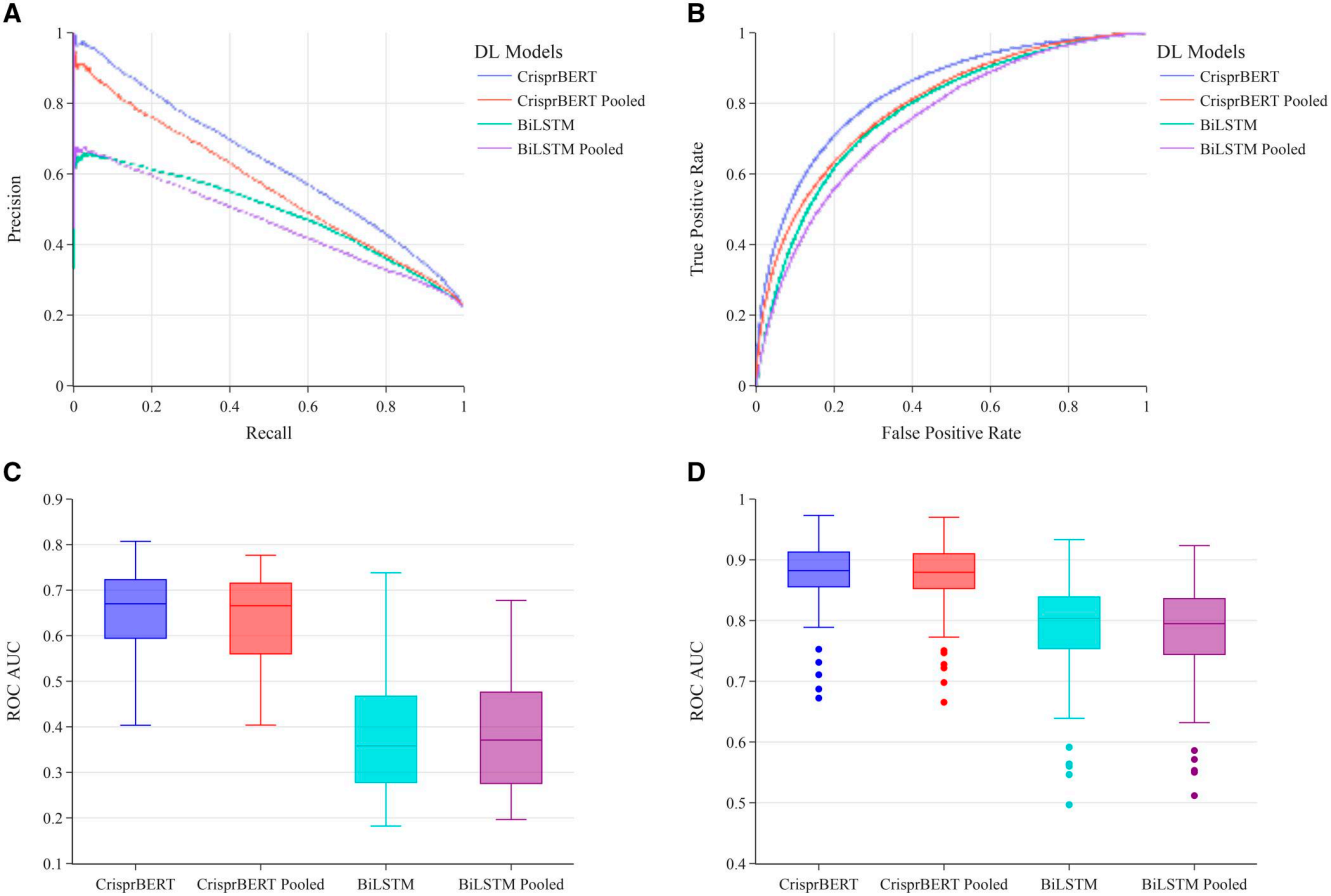
Average performance of leave-sgRNA-out test on pooled dataset (CHANGE-Seq + DeepCRISPR) against separate CHANGE-Seq dataset. (A) PR curve for the global performance of CripsrBERT and BiLSTM models trained on pooled datasets and trained on CHANGE-Seq dataset only. (B) ROC curve for the DL models as in (A). (C) Box-plot for the PR-AUC scores on each sgRNA for the same models as in (A). (D) Box-plot for the ROC-AUC scores on each sgRNA for the same models as in (A).

We observed the pooling strategy did not increase the performance of the models on this dataset. For the pooled dataset, the ROC-AUC and PR-AUC scores were 0.871 and 0.637 for the CrisprBERT model, compared with 0.821 and 0.401 for the BiLSTM model. For the individual dataset, the ROC-AUC and PR-AUC results were 0.881 and 0.653 for the CrisprBERT model and 0.876 and 0.412 for the BiLSTM model (Fig. 6A and B). When checking the performance of each individual sgRNA, models with both strategies performed similarly except the BiLSTM reduced performance slightly when trained on pooled datasets, particularly for the ROC-AUC performance (Fig. 6C and D). The PR-AUC for the pooled dataset does not differ much from the individual dataset in this case. For CrisprBERT, most sgRNAs had expected precision accuracy scores between 0.6 and 0.7.

#### 3.3.2 DeepCRISPR dataset

**Figure 7.**
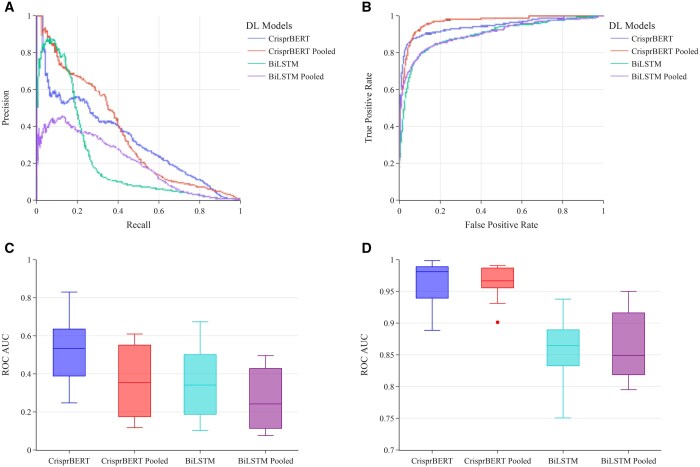
Average performance of leave-sgRNA-out test on pooled dataset (CHANGE-Seq + DeepCRISPR) against separate DeepCRISPR dataset. (A) PR curve for the global performance of CripsrBERT and BiLSTM models trained on pooled datasets and trained on DeepCRISPR-Seq dataset only. (B) ROC curve for the DL models as in (A). (C) Box-plot for the PR-AUC scores on each sgRNA for the same models as in (A). (D) Box-plot for the ROC-AUC scores on each sgRNA for the same models as in (A).

The results for the DeepCRISPR dataset were slightly different from the CHANGE-Seq dataset. Although the effect of pooling does not seem to improve the overall performance, we observed drastically increased ROC-AUCs with the pooling strategy for both models but not the PR-AUCs, where RP-AUC scores decreased slightly. Meanwhile, we observed that the average ROC-AUCs and PR-AUCs per sgRNA showed higher values than the global ROC-AUCs and PR-AUCs, largely because the number of positive off-target pairs is relatively small for many sgRNAs. Specifically, the global PR-AUC scores for the CrisprBERT model were 0.486 and 0.379 for the individual and pooled datasets, respectively. The global PR-AUC scores for the BiLSTM model were 0.385 and 0.355, again for the individual and pooled datasets, respectively (Fig. 7A). Accordingly, the global ROC-AUC scores for the CrisprBERT model were 0.960 and 0.972 for the individual and pooled datasets, respectively. Comparatively, the ROC-AUC scores for the BiLSTM model were 0.860 and 0.889 for the individual and pooled datasets, respectively (Fig. 7B). In addition, both models performed similarly with respect to the average performance on each sgRNA, with CrisprBERT doing slightly better than the BiLSTM model for both individual and pooled datasets (Fig. 7C and D).

## 4 Discussions

Accumulated experimental data have demonstrated that the CRISPR-Cas9 system induced DSB repair outcome is non-random (van Overbeek *et al.* 2016). Their off-target effects primarily depend on the properties of the endonuclease and the sgRNA sequences as well as the functional state (e.g. open chromatin regions) of the target genome (Hsu *et al.* 2013, Wang *et al.* 2014, Xu *et al.* 2015, Doench *et al.* 2016, 2014, Hanna and Doench 2020). These features provide an opportunity for building *in silico* models to predict the outcomes of designed sgRNAs, thus facilitating the optimized design of sgRNAs beforehand. In this study, a new sequence-based doublet stack encoding for sgRNA–DNA pairs was proposed to mimic the local energy configuration of Cas9 binding. Previous studies have highlighted the significance of mutations at specific positions within sgRNA–DNA pairs in influencing the specificity of the CRISPR-Cas9 system. In this study, we intend to conduct a similar analysis but focus on the distribution of stack doublets. Our results demonstrated a high degree of consistency in doublet occurrence across two independent datasets (i.e. CHANGE-Seq and DeepCRISPR datasets). Moreover, these doublets tend to co-localize with regions identified in earlier studies, indicating that doublet encoding may effectively capture biologically relevant information.

Compared to traditional single nucleotide-based encoding, the doublet stack encoding provides more potential vocabularies for downstream deep feature embedding and provides more flexibility to train a deep learning architecture-based model. Meanwhile, although triplet encoding expands the vocabulary from 256 to 4096, offering greater flexibility for model training, it also increases the challenge of training the model with a limited dataset. Therefore, doublet encoding provides a balance between the size of the training dataset and the model’s flexibility. In the CripsrBERT model, the BERT embedding approach was used to learn the deep representation of the doublets. Unlike the conventional word-to-vector embedding method used in the BiLSTM model, which generates fixed embeddings for each doublet regardless of its context, the embedding approach used in CrisprBERT is a contextualized doublet embedding model. It takes into account the surrounding doublets or sequences and their order when generating the doublet representations. Given the same doublet would be observed at different positions of the sgRNA–DNA pair and they might present different preferences in positive off-target sgRNA–DNA pairs, this contextual understanding allows CrisprBERT to capture the meaning of a doublet in different positions, which can further be beneficial for predicting the off-target effects of sgRNAs by considering the entire sgRNA–DNA sequence. Although this study mainly focused on CRISPR-Cas9 off-target prediction, the stacking encoding and the BERT embedding, as well as the BiLSTM architecture, could be applicable to CRISPR-Cas9 on-target activities prediction.

Sequence-based models are still demanding although it was reported additional epigenomic features or gene expression network features, which reflect the contexts of the editing sites could further improve the prediction. The sequence-only based models are particularly important when an additional epigenomic feature or gene expression network features data for the specific cell lines or primary cells (i.e. CHANGE-Seq data) are not available. Although the original Attention_to_mismatch was trained on sequence information and cell-type-specific gene properties derived from biological network and gene expression profiles, we were able to train the Attention_to_mismatch model with sequence information only. CRISPR-Net was an innovative approach for quantifying the CRISPR off-target activities but, in principle, is a sequence-based approach. These two models achieved comparable performances when conducting independent testing. The DeepCRISPR model integrated epigenetic and sequence features together and applied the autoencoder method to get a pre-trained feature representation. This might be informative to capture the potential sgRNA–DNA binding contexts from massive unlabeled pairs, which further benefits the prediction of the on-target and off-target effects of unknown sgRNAs. However, it performed worse in our study for the independent testing when predicting the sgRNA off-target effects from unused cell lines. One potential reason is that the epigenomics features we extracted from the ENCODE HepG2 cell line were the closest to but not perfectly measured to the profiles of the primary CD4+/CD8+ T cells from a healthy adult donor in the CHANGE-Seq dataset. Nevertheless, the cell-type-specific chromatin contexts, including epigenomic and gene expression data, do provide additional information for distinguishing different off-target activities and would be beneficial for building predictive models. Moreover, the physiochemical properties of nucleotides, structure, or energy-based features might further benefit the classifier construction. Incorporating the contexts-based features and structure features with the sequences features into a deep learning architecture would be a direction worth exploring.

Similar to Xiang *et al.* (2021), the advance in CRISPR sgRNA off-target prediction is mostly data-driven, rather than model-driven. This is partially due to the limited training dataset we currently have. Most of the advanced deep learning models require thousands of millions of parameters of the models to show the advantages. One limitation we acknowledge is the modest performance improvement achieved by the CrisprBERT. However, with more datasets becoming available, BERT-like embedding and models would be better to capture the essential DNA–RNA mismatch pairs, ultimately enhancing off-target detection. Meanwhile, most of the published deep learning models are trained on the DeepCRISPR dataset, which contains off-target pairs of only 30 sgRNAs (i.e. 18 sgRNAs from HEK293 and 12 sgRNAs from K562) or subsets of the DeepCRISPR dataset. In this study, we expanded the training dataset up to 140 sgRNAs by incorporating the most recent 110 sgRNAs from human primary cells in the CHANGE-Seq data. However, the preliminary exploration results indicated that simply combining the two datasets did not necessarily show a significant improvement in performance. We noted that the two datasets were generated by different protocols and that the CHANGE-Seq dataset showed many more positive pairs for each sgRNA than the DeepCRISPR dataset. How to integrate different datasets from different cell-lines, different platforms, and even different species would be an important question in the field. The CrisprBERT model developed in this study was not pre-trained. It was trained from scratch to achieve an effective embedding of the input sgRNA–DNA sequence pairs. Besides the potential parameters regarding the embedding dimension, we adopted other default settings to train a BERT model from scratch. For the details, users are referred to the original documents provided by the HuggingFace team (Wolf *et al.* 2020). Potentially, a BERT-like model can be pre-trained on biological “vocabulary” and “sentences.” This, however, will require biological context-specific tasks, compared to those that are used to train the current BERT-like models in the NLP field. Moreover, heterogeneous data integration, data augmentation, and effective transfer learning strategies might be helpful to pre-train the BERT model and to learn deep feature representations.

## Supplementary Material

vbae184_Supplementary_Data

## Data Availability

The code for CrisprBERT and the associated datasets are available at GitHub: https://github.com/OSsari/CrisprBERT.
