# Supplementary material for: Predicting CRISPR-Cas9 off-target effects in human primary cells using bidirectional LSTM with BERT embedding
Source: Bioinform Adv. 2024 Dec 30;5(1):vbae184. doi: 10.1093/bioadv/vbae184 (PMC11696696; doi:10.1093/bioadv/vbae184)
Supplement: vbae184_Supplementary_Data [file vbae184_supplementary_data.docx]

**Predicting CRISPR-Cas9 off-target effects in human primary cells using bidirectional LSTM with BERT embedding**

Orhan Sari^1^, Ziying Liu^2^, Youlian Pan^2*^, Xiaojian Shao^2,3*^

^1^Department of Mining and Materials Engineering, McGill University, Montreal, QC, Canada, ^2^Digital Technologies Research Center, National Research Council Canada, Ottawa, ON, Canada, ^3^Ottawa Institute of Systems Biology, Department of Biochemistry, Microbiology and Immunology, University of Ottawa, Ottawa, ON, Canada

*To whom correspondence should be addressed.

Supplementary Materials


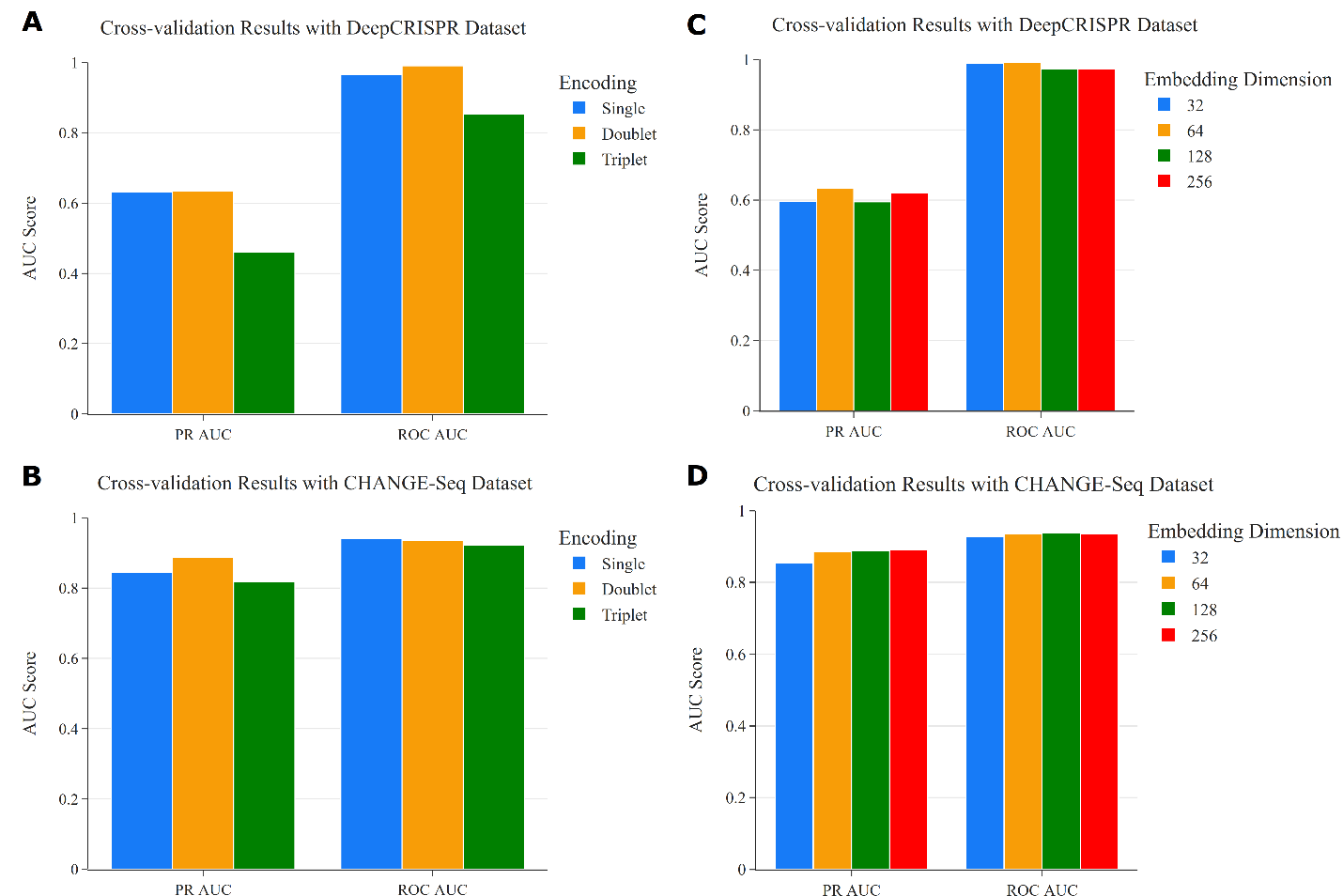


**Figure S1. Cross-validation performance of crisprBERT model on different hyperparameter settings.** Cross-validation performance of crisprBERT on different encoding of the sgRNA-DNA pairs with DeepCrispr dataset (A) and CHANGE-Seq dataset (B). Cross-validation performance of the models with different embedding dimensions at the BERT layer for DeepCRISPR dataset (C) and CHANGE-Seq dataset (D).


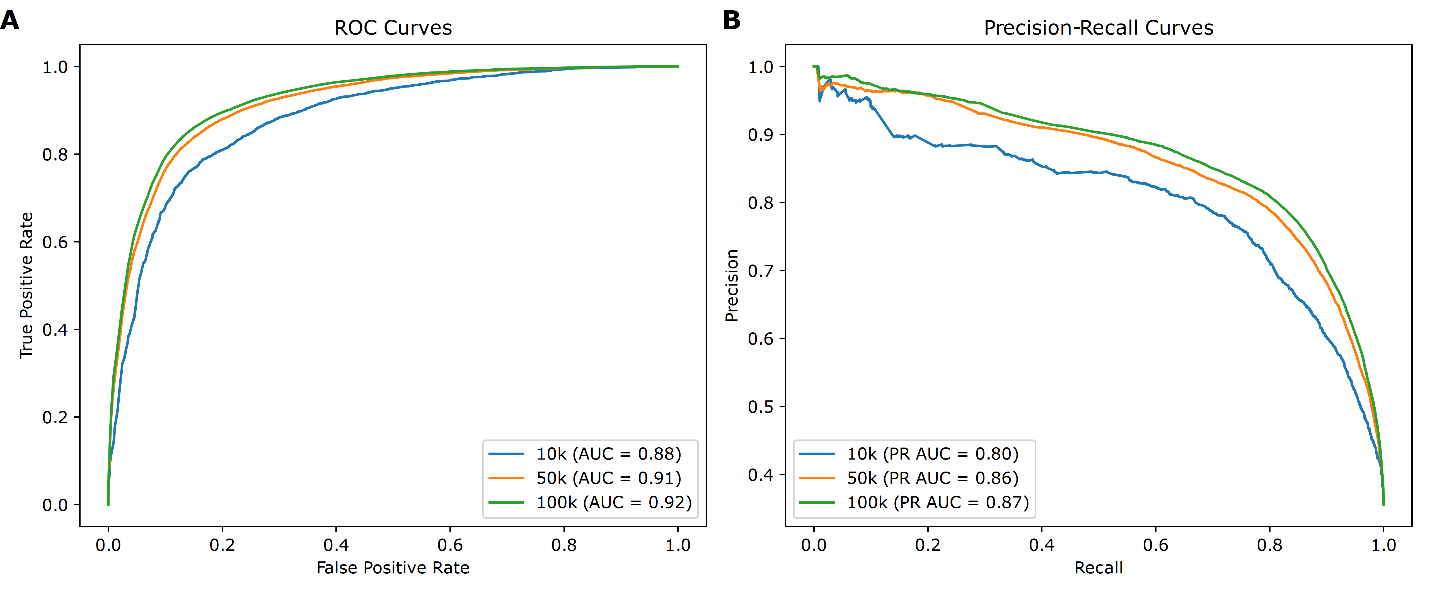


**Figure S2. Cross-validation performance of crisprBERT model on different training data sizes.** Cross-validation was conducted on the subsets of the CHANGE-Seq dataset with data sizes of 10k, 50k and 100k. Subset of the CHANGE-Seq dataset was randomly extracted. It demonstrated the performance (in terms of both ROC AUC and PR AUC) increases when the dataset size increases.


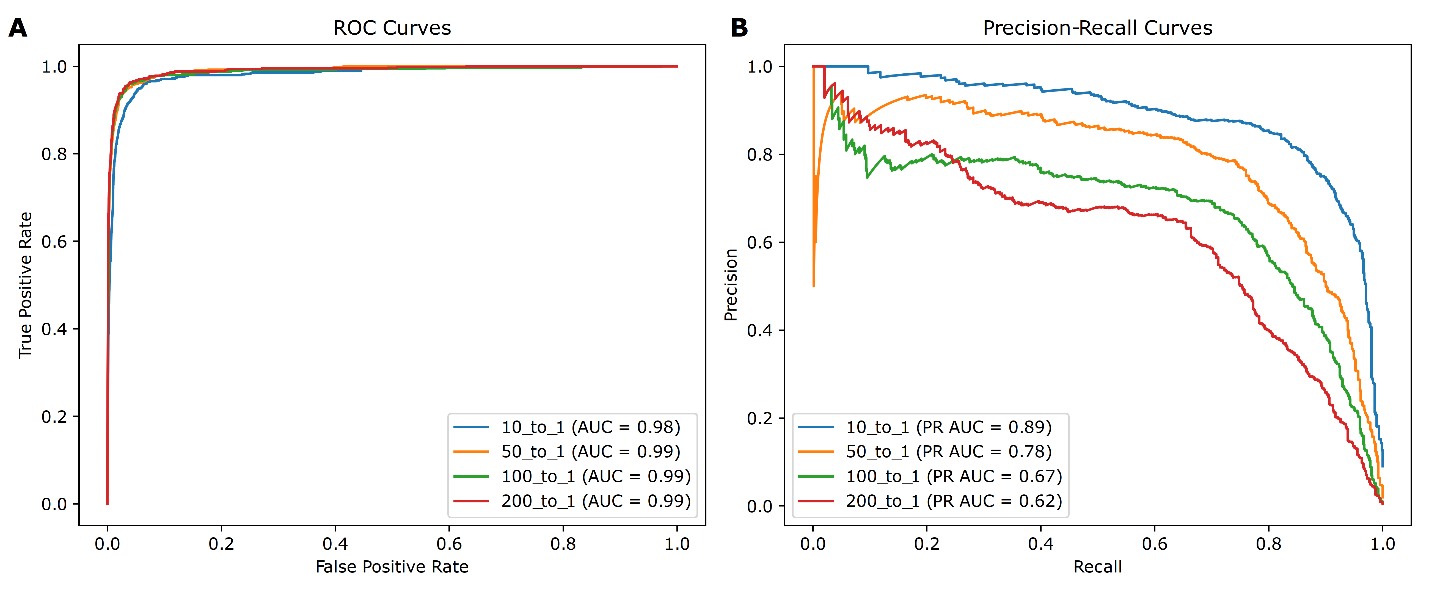


**Figure S3. Cross-validation performance of crisprBERT model on a dataset with different imbalance ratios.** The ratio of negative pairs to positive pairs ranged from 10 to 1, 50 to 1, 100 to 1 and 200 to 1 were applied to the DeepCRISPR dataset through over-sampling the positive pairs. Cross-validation was then conducted on these datasets with different imbalance ratios. It demonstrated that PR AUC increases when the data imbalance ratio decreases while ROC AUC remains almost unchanged.


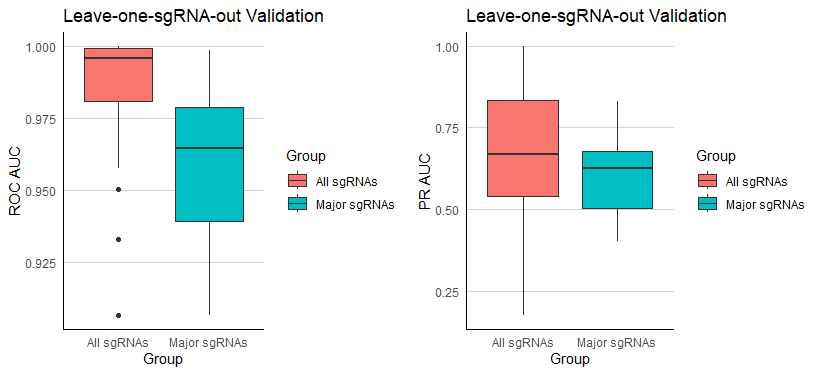


**Figure S4**. **Performance of leave-one-sgRNA-out validation on the DeepCRISPR dataset**. The distributions of ROC AUC (left) and PR AUC (right) across all sgRNAs or major sgRNAs (i.e. sgRNAs with >=30 positive pairs) are presented in the boxplots. The average ROC AUC is 0.98 and the PR AUC is 0.66 for all sgRNAs. When limiting to sgRNAs with 30 or more positive pairs, the average ROC AUC and PR AUC become 0.96 and 0.60, respectively.
